# Influencing Factors, Formation Mechanism, and Pre-control Methods of Coal Miners′ Unsafe Behavior: A Systematic Literature Review

**DOI:** 10.3389/fpubh.2022.792015

**Published:** 2022-03-07

**Authors:** Li Yang, Xue Wang, Junqi Zhu, Zhiyuan Qin

**Affiliations:** School of Economics and Management, Anhui University of Science and Technology, Huainan, China

**Keywords:** behavioral interventions, coal miners, coal mine safety management, formation mechanism, unsafe behavior

## Abstract

Coal mine accidents are mainly caused by the unsafe behavior of workers. Studying workers' unsafe behaviors can help in regulating such behaviors and reducing the incidence of accidents. However, there is a dearth of systematic literature review in this area, which has hindered mine managers from fully understanding the unsafe behavior of workers. This study aims to address this research gap based on the literature retrieved from the Web of Science. First, a descriptive statistical analysis is conducted on the year, quantity, publications, and keywords of the literature. Second, the influencing factors, formation mechanism, and pre-control methods of coal miners' unsafe behavior are determined and discussed, and the research framework and future research directions of this study are proposed. The study results will help mine safety managers fully understand the influencing factors, formation mechanism, and pre-control methods of workers' unsafe behavior, and lay a theoretical foundation for the future research direction in this field.

## Introduction

Coal is one of the most critical energy sources globally and occupies an essential global energy structure. However, the frequent occurrence of coal mine accidents has seriously affected the safe mining of coal. According to statistics, accidents caused by human factors account for more than 90% of all coal mine accidents, and these human factors refer to various unsafe behaviors of workers (1–3). Considering that workers' unsafe behavior is the leading cause of coal mine accidents, it is necessary to adopt complementary intervention strategies to control workers' unsafe behavior in practice. Scholars have carried out a lot of exploration and research in this area (4–6).

The unsafe behaviors of coal miners refer to those behaviors that miners fail to strictly abide by the safety rules and regulations in the production process, which may negatively affect organizational and personal safety (7, 8). The formation of coal miners' unsafe behavior is complicated and involves many factors, which prompts scholars to discuss the influencing factors of coal mine workers' unsafe behavior from different angles. Yu et al. (9) used ANP (Analytic Network Process) and SD (System Dynamic) model to analyze the influence of individual factors, group factors, physical environment, safety leadership, and safety management factors on the unsafe behaviors of coal miners, and ranked their importance. Liu et al. (10) also analyzed the types of factors influencing coal miners' unsafe behaviors and pointed out the significance of these factors. Li et al. (11, 12) studied the relationship between safety attitudes and safety behavior in the coal mine industry, and found that strengthening safety education and training for miners could improve their unsafe behavior and prevent accidents. They also proposed a mixed recommendation method that could improve the effectiveness of safety training. In addition, scholars have adopted different research theories and methods to investigate the formation mechanism of coal miners' unsafe behaviors, such as SEM (Structural Equation Model), SD model, and game theory model. Given the necessity of adopting intervention strategies to control coal mine workers' unsafe behaviors, scholars also discussed the pre-control methods of coal mine workers' unsafe behaviors from different angles.

Although there are an increasing number of studies on the unsafe behavior of coal miners, there is still a lack of relatively complete and systematic research framework. To fill the gap, this study used a systematic analysis method to summarize and analyse the historical literature of coal miners' unsafe behavior. The focus of the literature review is to study the influencing factors, formation mechanism, and pre-control measures of unsafe behavior of coal miners. The research results of this paper will help the managers of coal mine enterprises to have a more comprehensive understanding of the unsafe behavior of miners to take effective management measures to control the dangerous behavior of workers. It will also help coal mine safety researchers lay a theoretical foundation for further research in the future.

This paper is divided into five parts. Section Literature Source and Analysis introduces the research methods and the literature analysis process in detail. Section Literature Review reviews the relevant literature from three aspects: the influencing factors, the formation mechanism, and the prevention and control measures of unsafe behavior of coal miners. Section Results and Discussion discusses the study results and proposes the future research directions. Finally, Section Conclusions summarizes the findings of this study while detailing what is lacking in the current research and how it can be addressed in the future research.

## Literature Source And Analysis

### Literature Source

**Figure 1:**
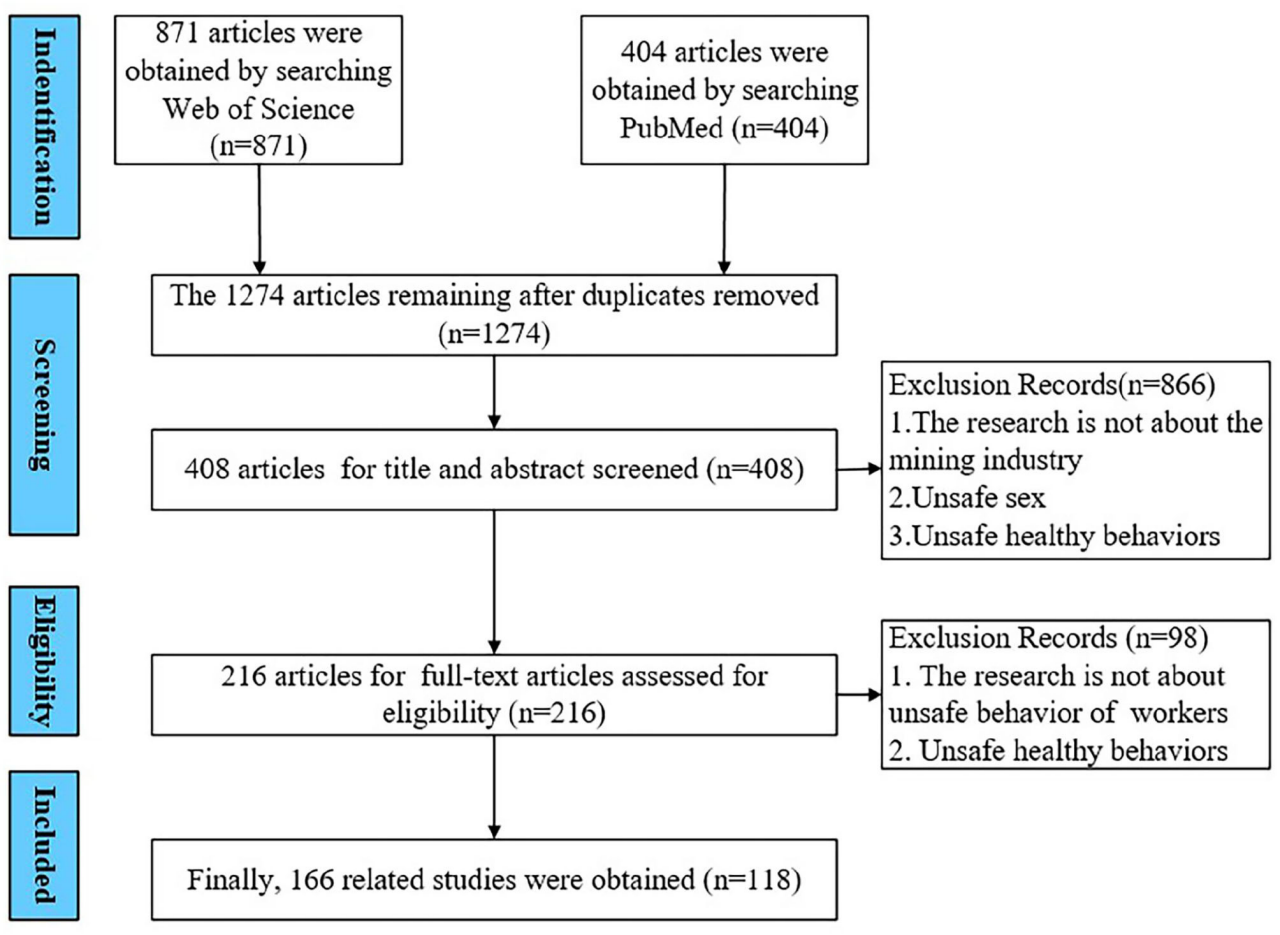
Flowchart of the systematic review process (PRISMA flow diagram).

The premise of literature review is the selection of appropriate keywords for searching the literature in the database (13). It is a summary of the existing research results, so the selected relevant literature must be retrieved and classified. This study takes the “Web of Science” core database and “Pubmed” as the source of literature retrieval, because they contain most of the literature published since 1900, including more than 12,000 leading journals in the world. After scrutinizing several studies, it found that although some articles do not have the term “coal mine workers” in the theme, the content is related to the unsafe behavior of coal miners. Therefore, to ensure the comprehensiveness of the literature searched, the search topic is extended from topic 1 “unsafe behavior of coal mine workers” or “human error of coal mine workers” or “unsafe behavior and coal mine safety” or “unsafe behavior and mining safety” or “human error and coal mine safety” or “human error and mining safety” to topic 2 “unsafe behavior of workers.” In addition, the time range of the literature search was set from January 1, 2007 to July 31, 2021.The literature types included articles and review articles, and the language was English. The Web of Science core database can be retrieved at: https://www.webofscience.com/wos/alldb/summary/a035bd83-b5e7-43e7-b842-e33f13b0baee-1d1eafb1/relevance/1, Scopus database search format is “((((((unsafe behavior of coal mine workers[Title]) OR (human error of coal mine workers[Title])) OR (human error[Title] AND coal mine safety[Title])) OR (human error[Title] AND mining safety[Title])) OR (unsafe behavior of workers[Title])) AND ((“2007/01/01” [Date - Publication]: “2021/07/31” [Date - Publication]))) AND (English[Language])”. Literature screening and inclusion criteria are shown in Table 1. After detailed combing, a document screening process was proposed to determine the papers that can be reviewed in this study, as shown in Figure 1. A total of 118 documents related to the research topic were screened.

**Table 1:** Literature inclusion and exclusion criteria.

**Description**	**Literature inclusion criteria**
Publication time	Between January 1, 2007 and July 31, 2021.
Document types	Articles or review articles.
Languages	Literature published in English.
Research methods	Developed to study the unsafe behavior of coal miners.
Research object	Coal miners, coal mine safety, and coal mine accidents.
Core research content	Influencing factors of coal mine workers' unsafe behavior, formation mechanism of coal miners' unsafe behavior, measures to control the unsafe behavior of coal miners, and application of information technology in the field of workers' unsafe behavior management.

### Literature Analysis

#### Literature Publication Year and Quantity Trend Analysis

**Figure 2:**
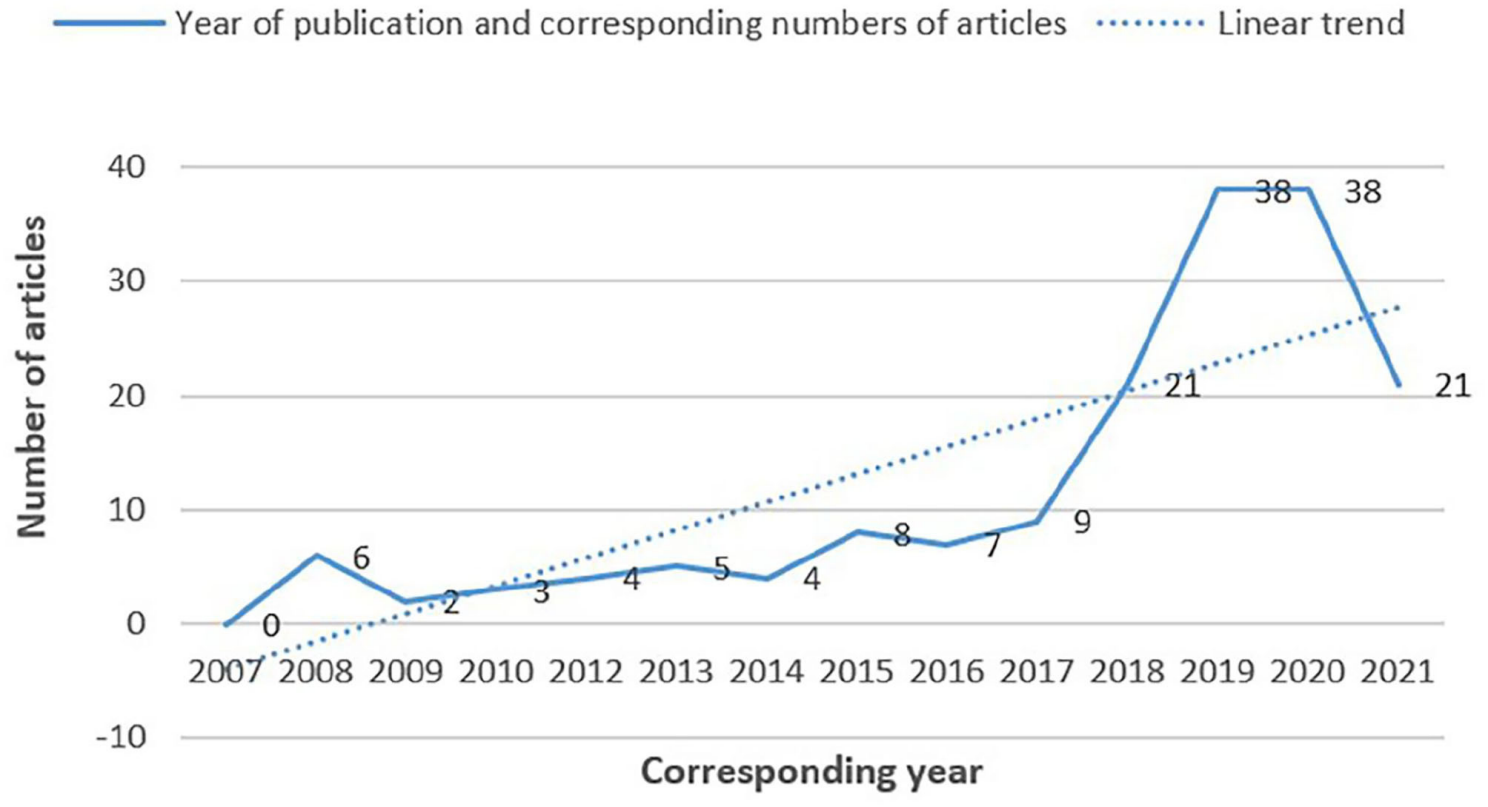
The number of publications in different years (2007–2021).

Figure 2 shows the number of publications in different years since 2007. As can be seen from the picture, before 2017, only a few articles on “unsafe behavior of coal mine workers” were published. After 2017, the number of published articles gradually increased, reaching 38 in 2019. This reflects that people have become increasingly interested in research on the “unsafe behavior of coal mine workers” in recent years. In addition, we can also see that after 2019, the research on “unsafe behavior of coal miners” has decreased, but compared with 2007, it still shows an overall upward trend. From these, we can know that 2007 is the initial stage of research on “unsafe behaviors of coal miners.” Various studies are not mature yet, and they are all in the exploration stage. With the increase in the number of articles published by scholars, by 2019, studies on the “unsafe behavior of coal miners” have gradually entered a mature stage, and scholars have studied this topic from various aspects. After 2019, as the primary research of this research subject matures, scholars are required to conduct more in-depth research on this topic, which also poses significant challenges for scholars. Given the above, future scholars should research “unsafe behaviors of coal miners” from a more in-depth and innovative perspective.

#### Literature Publication Source Analysis

**Figure 3:**
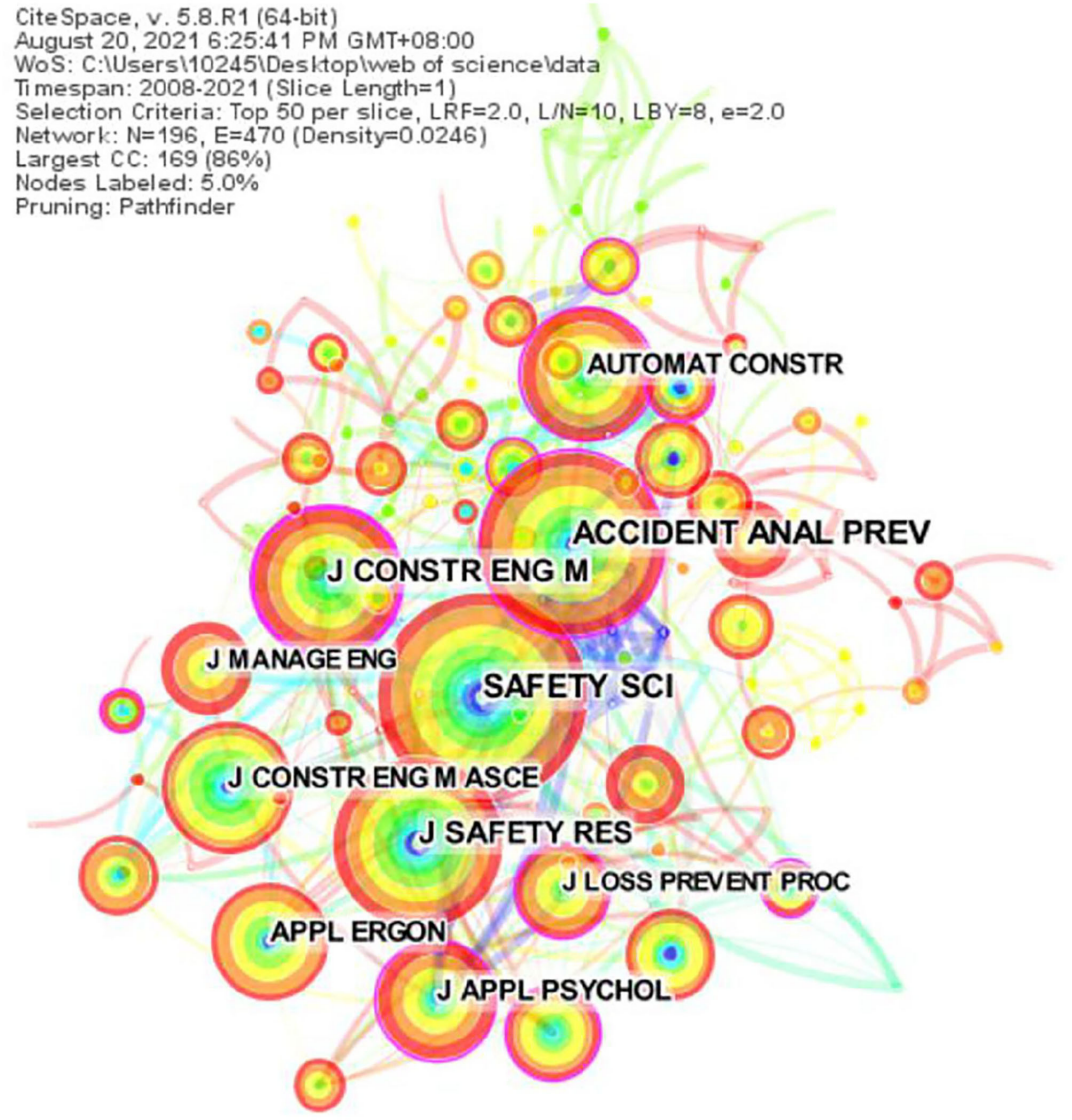
The result of literature publication source analysis.

Figure 3 shows the source publications using the visual analysis tool CiteSpace. A circle represents a publication, and the larger the circle, the greater the number of papers published in that publication. This study found that most papers were published in “Risk and safety Management” (e.g., Safety Science, Accident Analysis & Prevention, and Journal of Safety Research) field and “Engineering Management” (e.g., Journal of Construction Engineering and Management, Automation in Construction) field. As can be seen from the figure, Safety Science is the most published Journal, followed by Accident Analysis & Prevention and Journal of Safety Research. Scholars can refer to the published literature of these journals in their future studies on the “unsafe behavior of coal miners.”

#### Keyword Cluster Analysis

**Figure 4:**
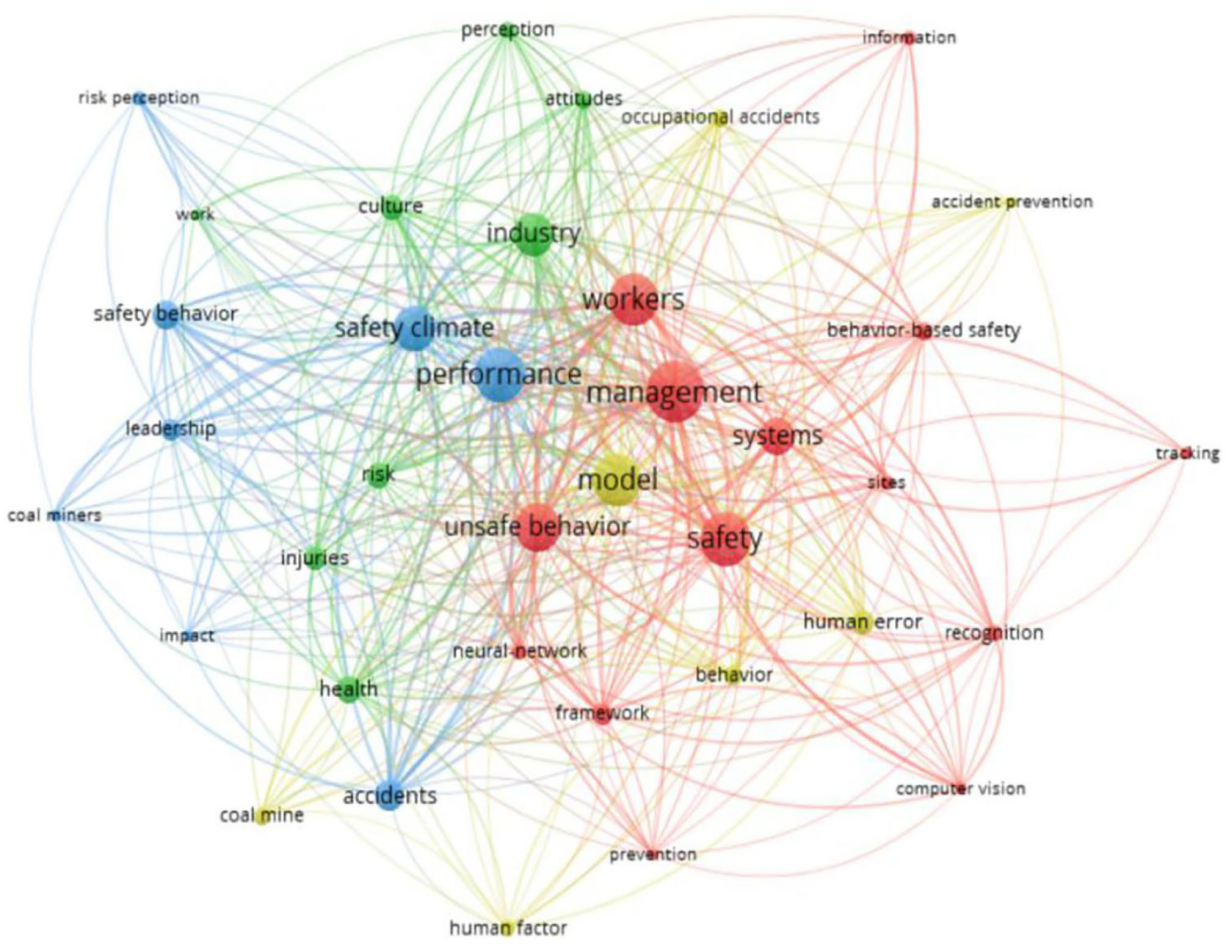
The cluster graph of keyword co-word analysis.

Keyword cluster analysis was conducted to identify key co-words in articles, which are often a research hotspot in the field. Therefore, this analysis can be used to identify the sub-themes explored by scholars in the field of “unsafe behavior of coal mine workers.” In this study, the association strength algorithm in VOSviewer software was used to identify 37 keywords with a frequency greater than or equal to 5, and all keywords were divided into five categories. Among the keywords identified by the software, some keywords were merged because of their similar meanings (for example, climate and safety climate can be integrated into safety climate, and safety management and management can be integrated into management). The merged keywords were divided into four categories, as shown in Table 2. Figure 4 shows the merged keyword clustering results and the relationship between the keywords and each set. Each color in the figure represents different sets, and the size of the nodes indicates the frequency of the keywords being quoted. The larger the node, the higher the frequency of the keywords being quoted. The distance between nodes indicates the strength of the relationship between them. The farther the distance, the weaker the relationship. As shown in Figure 4, the core keywords of this study (the largest circle of each color) include “management,” “model,” “performance,” and “industry.” These core keywords reflect the research content of this study (e.g., improving safety performance and strengthening management). The keywords around them are the influencing factors and specific measures affecting this core content, which further proves that the research direction of this study is to systematically analyze the collected literature to determine the influencing factors, formation mechanism, and pre-control measures of coal miners' unsafe behaviors.

**Table 2:** The clustering results of keywords co-word analysis.

**Classification**	**Keywords**
**Cluster 1**	Behavior-based safety, computer vision, framework, information, management, neural-network, prevention, recognition, safety, sites, systems, tracking, unsafe behavior, workers.
**Cluster 2**	Attitudes, culture, health, industry, injuries, perception, risk, work.
**Cluster 3**	Accidents, coal miners, impact, leadership, performance, risk perception, safety behavior, safety climate.
**Cluster 4**	Accident prevention, behavior, coal mine, human error, human factor, model, occupational accidents.

## Literature Review

**Figure 5:**
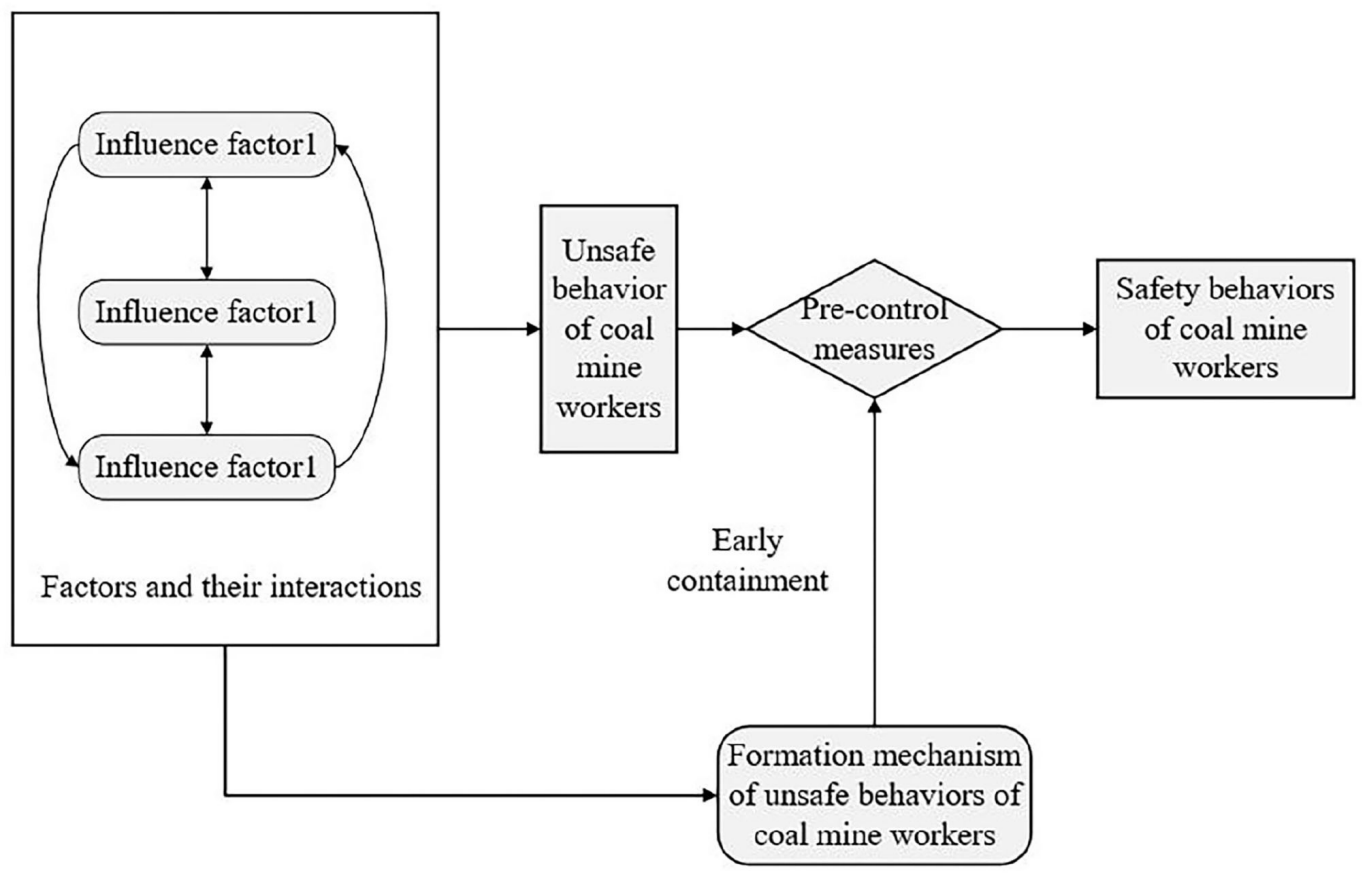
The research framework in the studied field.

Cao et al. (4) believed that it is necessary to thoroughly analyze the causes of coal mine accidents to help managers implement effective safety management measures to improve the performance of the entire coal mine safety production process. Summarizing the research results in the field of unsafe behavior of coal miners, we found that the three sub-themes of influencing factors, formation mechanism, and pre-control measures of unsafe behavior of coal miners have been discussed extensively in history. On this basis, the present study proposes the research framework of this research field (Figure 5).

### Influencing Factors of Coal Miners′ Unsafe Behavior

To explore the main influencing factors of coal miners' unsafe behavior, scholars have conducted several studies from different perspectives. Seo et al. (14) pointed out that individual characteristics, work pressure, safety culture, safety climate, and other factors have different degrees of influence on workers' unsafe behaviors. Tong et al. (15) also studied the influencing factors of miners' unsafe behaviors from the perspective of behavior, indicating that the unsafe behaviors caused by improper operation, distraction, ignoring safety warnings, improper equipment use, and risky behaviors of workers are the main causes of coal mine gas accidents. Liu et al. (10) established a coal mine human factor analysis and classification system to analyze the types of factors affecting miners' unsafe behaviors, suggesting that the external environment, unsafe leadership, preconditions of unsafe behaviors, and organizational influence are the main types of factors affecting coal miners' unsafe behaviors. Based on a summary of research results, Yu et al. (9) identified the main influencing factors of coal miners' unsafe behaviors from five aspects: individual factors, physical environment, safety leadership, safety management, and group factors. Fu et al. (16) reported that the lack of safety knowledge, habitual illegal behavior, improper operation, and other reasons caused by miners' unsafe behavior are the main causes of accidents in coal mines. In view of the summary of scholars' historical research results and the actual situation of coal mine safety management, this study classifies the factors influencing coal miners' unsafe behavior in more detail from three aspects: individual factors, organizational management factors and environmental factors.

#### Individual Factors

This level focuses on individual of coal miners, such as their individual characteristics, mental status, and physiological status, as shown in Table 3.

**Table 3:** Individual factors.

**Major categories**	**Category segmentation**	**Detailed categories**	**Source**
Individual factors	Individual characteristics	Individual age	Wang et al. (17), Nouri et al. (18)
		Work experience	Chen et al. (19), Wang et al. (17), Nouri et al. (18)
		Personality traits	Baba et al. (20), Sing et al. (21)
	Psychological factors	Safety awareness	Yu et al. (9), Wang et al. (22)
		Safety attitude	Li et al. (11), Tam et al. (23), Li et al. (24), Li et al. (25)
		Work stress	Seo et al. (14), Wu et al. (26)
		Risk perception	Chen et al. (27), Man et al. (28)
	Physiological factors	Fatigue	Kapp et al. (29), Duma et al. (30), Fang et al. (31), Ren et al. (32), Duan et al. (33)
		Insomnia	Kao et al. (34)

The individual characteristics influencing workers' unsafe behavior mainly include individual age, work experience and personality traits. Owing to the different research perspectives of scholars, different conclusions may be drawn regarding the influence of the same individual characteristics on unsafe behaviors. For example, Seo et al. (14) identified that personal characteristics have an indirect influence on safety behavior. Chen et al. (17–19) also reported that individual characteristics, such as gender, age, education level, and working years, have a significant impact on employees' behaviors: inexperienced and younger coal miners are more likely to engage in unsafe behavior. In contrast, Malakoutikhah et al. (35) asserted that workers' age, education level, work experience, and years of study had no influence on their unsafe behavior. Contrary to previous studies, Paul et al. (36) found that the age of employees was significantly positively correlated with work-related accidents among employees while the length of service was not correlated with work-related accidents among employees. Some scholars have also studied the influence of workers' personality traits on unsafe behaviors. For example, Baba et al. (20, 21) identified that there was a significant relationship between workers' personality and unsafe behaviors, and workers' proactive personality would greatly promote their safety performance and avoid the occurrence of unsafe behaviors.

The mental status of individual workers can also influence their unsafe behaviors, which mainly include safety attitude, safety awareness, work pressure and risk perception. Yang et al. (37, 38) reported that the main cause of coal mine safety accidents was the unsafe behavior of miners affected by emotions. When workers are in a bad mental state, they are prone to unsafe behaviors. Scholars have studied the influence of different mental indicators on workers' unsafe behavior. For example, Li et al. (11, 23–25) found that the safety attitude of coal miners has a positive impact on safety behavior, safety attitudes of employees will significantly affect safety performance, and a good safety attitude can improve the safety performance of the entire organization. Wu et al. (26) identified that there is a negative correlation between workers' work stress and safe behaviors. Guo et al. (39, 40) also pointed out that production pressure is a key factor affecting workers' safety motivation, safety knowledge, safety participation and safety compliance. Wang et al. (22) pointed out that there was a positive correlation between individual safety awareness and safety behavior, and safety climate positively moderated the relationship between them. In addition, Man et al. (27, 28) corroborated that workers' outcome expectation, risk perception (worry and insecurity), and attitude toward risky behaviors also had a certain impact on their unsafe behaviors.

Workers' physiological status can also affect their unsafe behaviors. Existing studies indicate that physiological abnormalities are an important factor affecting workers' unsafe behaviors (41). For example, Kapp et al. (29–31) reported that fatigue has a negative impact on workers' safety performance, workers are more prone to make mistakes when exhausted, and fatigue of coal miners is significantly related to coal mine accidents. Duan (33) identified that muscle fatigue is an important cause of musculoskeletal injury and can easily induce unsafe behaviors. Ren et al. (32) also found that with the increase in the physical consumption of coal miners, their enthusiasm for work and work efficiency declined. In addition, Kao et al. (34) identified that insomnia would lead to a reduction in employees' safety behaviors, which would further lead to industrial accidents. Leung et al. (42) also asserted that job certainty, colleague support, and safety equipment can predict workers' physiological stress, and safe behavior can reduce accident risk. In response to the effects of these physiological status, Jing et al. (43) constructed a safety early warning system, which can measure physiological data of miners in real time, judge their health status, and give early warning, thus providing an effective way to reduce the occurrence of accidents.

#### Organizational Management Factors

Organizational management factors mainly include organizational influence, safety management and safety leadership, among which safety management and safety leadership are management factors, as shown in Table 4.

**Table 4:** Organizational management factors.

**Major categories**	**Category segmentation**	**Detailed categories**	**Source**
Organizational management Factors	Organizational influence	Safety climate	Hofmann et al. (44),Casey et al. (45), Casey et al. (22)
		Safety culture	Kagan et al. (46), Harsini et al. (47)
		Communication	Yu et al. (9), Liao et al. (48)
	Management factors	Safety management	Choudhry et al. (49), Lv et al. (50), Zhang et al. (51), Tong et al. (52)
		Safety leadership	Zohar et al. (53), Jiang et al. (54), Bonsu et al. (55), Oah et al. (56)

In a complex industrial system, the completion of a job requires the cooperation of multiple employees, so their behavior will also be affected by the organization. The organizational influence factors that affect workers' unsafe behavior mainly include safety climate, safety culture and communication. Scholars have carried out a very rich research in this area. For example, Aliabadi et al. (57) found that organizational deficiencies were the main cause of accidents in the mining sector and had a direct positive impact on employee violations and errors. Hofmann et al. (22, 44, 45) reported that the organizational safety climate affects individual safety behavior and safety awareness. Kagan et al. (46, 47) also identified that safety culture in organizations can have a positive impact on workers' safety behaviors and effectively reduce work errors. In addition, Liao et al. (48) pointed out that increasing the frequency of communication between managers and employees and adding additional communication channels can effectively improve the safety climate of an organization. Li et al. (58, 59) also asserted that the interaction, communication, and trust among organization members can help improve employees' unsafe behaviors and effectively reduce the occurrence of accidents. In view of the above organizational impact, Chen et al. (27, 28) established that the successful implementation of organizational safety culture and attitude, organizational safety promotion policies, and safety education and training can effectively improve workplace safety production and workers' safety behaviors.

Safety management also has an important impact on the unsafe behavior of coal miners, and it has been studied from different perspectives. For example, Choudhry et al. (49) confirmed that organizational management played an important role in improving workers' unsafe behaviors. Lv et al. (50) also found that the implementation of behavioral safety management can effectively reduce the occurrence of accidents caused by the unsafe behaviors of coal miners. In addition, Zhang et al. (1, 51) analyzed the impact of a series of safety management measures on workers' safety performance, pointing out that safety education and training are the most effective safety management measures, and the implementation of safety education and training can improve workers' unsafe behaviors and reduce the occurrence of accidents. Tong et al. (52) also reported that behavioral intervention can reduce the occurrence of unsafe behaviors of coal miners and proposed a targeted intervention method to intervene in the unsafe behaviors of coal miners.

The safety leadership of managers in organizations also has an impact on workers' unsafe behaviors. For example, Zohar et al. (53) found that supervisory leadership can act as a gatekeeper, while transformational leadership provides better protection. They point out that leaders' behavioral interventions and regulatory measures can significantly improve an organization's safety climate and workers' behavioral safety performance. Jiang et al. (54) also studied the relationship between different leadership behaviors and workers' unsafe behaviors, and identified that negative leadership behaviors are significantly negatively correlated with miners' safety behaviors. In addition, Bonsu et al. (55) asserted that insufficient leadership was the most common systemic factor leading to accidents. They point out that managers' leadership behavior has a significant impact on employees' unsafe behavior and serves as a model for employees' behavior. Oah et al. (56) reported that safety leadership and safety climate had a negative impact on cognitive and emotional risk perception, and workload, safety leadership, and safety climate had a greater impact on perceived risk than accident experience, especially emotional risk perception.

#### Environmental Factors

Environmental factors affecting coal miners' unsafe behavior mainly include physical environment and social environment. Physical environment mainly refers to temperature, humidity, noise, etc., while social environment mainly refers to interpersonal relationship, social identity, social cognition, etc., as shown in Table 5.

**Table 5:** Environmental factors.

**Major categories**	**Category segmentation**	**Detailed categories**	**Source**
Environmental factors	Physical environment	Physical climate	Glazner et al. (60), Chi et al. (61)
		Terrain	Glazner et al. (60)
		Noise	Westaby et al. (62), Chen et al. (63)
	Social environment	Social interaction	Schwatka et al. (64), Westaby et al. (62)
		Workplace exclusion	Chen et al. (63)
		Social psychological safety climate	Mohamed et al. (65), Mohammadfam et al. (8), Huang et al. (66)

Physical environment refers to the physical factors in the working environment of the coal mine, mainly including weather, terrain, noise and other natural factors. In view of physical environmental factors, scholars have carried out research from different angles. For example, Harsini et al. (67) pointed that unsafe working conditions can lead to unsafe behaviors of workers. Glazner et al. (60) reported that weather, terrain, and poor lighting were the main causes of industrial accidents. Chi et al. (61) also found that working conditions (such as working face conditions and weather) were closely related to workers' behaviors, accident types, and the degree of accident injuries through the analysis of safety accidents in doctoral dissertations in the United States during 2002–2011. Yoon et al. (68, 69) pointed out that a strong noise environment seriously affects the physical and mental health of operators and leads to unsafe behaviors of workers. On the contrary, Lu et al. (70) proposed that pleasant sounds can help workers learn safe behaviors and effectively reduce unsafe behaviors.

Social environment mainly refers to the internal and external environment of the organization where workers are, mainly refers to social interaction, workplace exclusion, social psychological safety climate and so on. Workers cannot avoid interacting with people around them in both work and life, and the quality of social interaction also has an important influence on their safety behaviors (64). For example, Westaby et al. (62) reported that the risk propensity of parents of young workers significantly affects the risk propensity of young employees, leading to work-related accidents among young workers. In addition, Chen et al. (63) pointed out that workplace exclusion has a significant impact on employees' psychological detachment and emotional exhaustion, which in turn affects employees' unsafe behaviors. Yu et al. (71) also demonstrated that a psychosocial safety climate could reduce miners' unsafe behavior through the mediating effect of job stress and job burnout. The internal environment of coal miners also has an impact on their unsafe behavior. For example, Mohamed et al. (65) reported that workers working in a more collective and higher uncertainty avoidance environment are more likely to have safety awareness and belief and can show safer on-site behaviors. Mohammadfam et al. (8) also showed that safety attitudes, safety knowledge, and supportive environment were the best predictors of worker safety behavior, and immediate improvement of supportive environment and employee participation is the best strategy to achieve a high proportion of safe behavior in the workplace. In addition, Huang et al. (66) asserted that a good safety knowledge transmission environment can promote safety behavior of field workers and the application and inspiration of safety knowledge.

**Figure 6:**
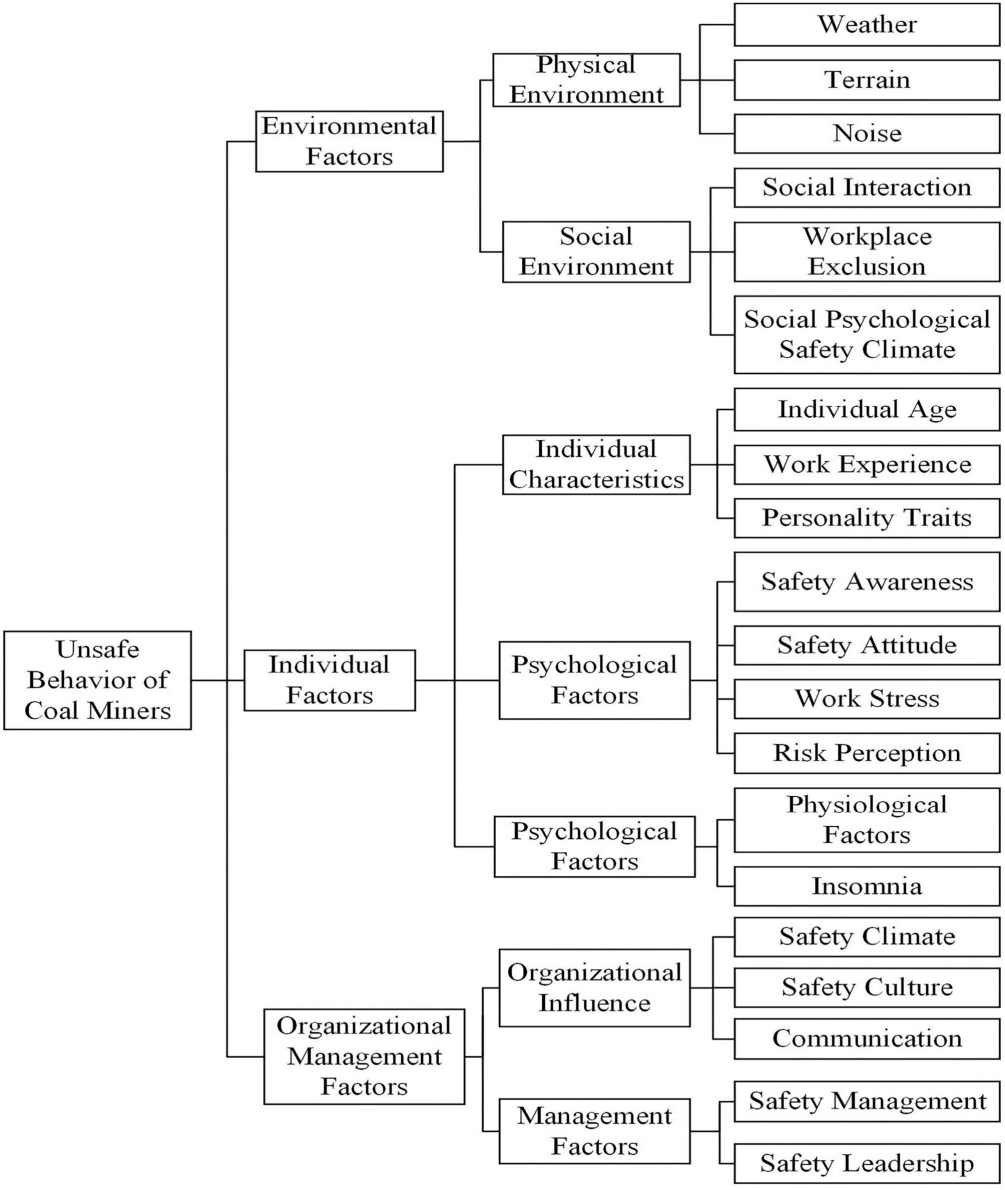
The index system framework of influencing factors of coal mine workers' unsafe behavior.

As seen from the above description, scholars' studies on the factors influencing coal mine workers' unsafe behavior vary from different angles. Although some scholars have studied the influencing factors of unsafe behaviors in coal mines systematically, the index systems constructed are also other. It can see that there is no unified index system standard in the field of systematic research on the influencing factors of coal mine workers' unsafe behaviors. Therefore, based on the previous research results and the actual production situation of coal mines, this study constructed the index system of influencing factors of coal mine workers' unsafe behaviors, as shown in Figure 6 and Table 6.

**Table 6:** Index system of influencing factors of coal mine workers' unsafe behavior.

**Target layer**	**Level 1 indicators**	**Level 2 indicators**	**Level 3 indicators**	**Source**
Factors influencing coal mine workers' unsafe behavior	Environmental factors	Physical environment	Weather	Glazner et al. (60), Chi et al. (61)
			Terrain	Glazner et al. (60)
			Noise	Westaby et al. (62), Chen et al. (63)
		Social environment	Social interaction	Schwatka et al. (64), Westaby et al. (62)
			Workplace exclusion	Chen et al. (63)
			Social psychological safety climate	Mohamed et al. (65), Mohammadfam et al. (8), Huang et al. (66)
	Organizational management factors	Organizational influence	Safety climate	Hofmann et al. (44), Casey et al. (45), Casey et al. (22)
			Safety culture	Kagan et al. (46), Harsini et al. (47)
			Communication	Yu et al. (9), Liao et al. (48)
		Management factors	Safety management	Choudhry et al. (49), Lv et al. (50), Zhang et al. (51), Tong et al. (52)
			Safety leadership	Zohar et al. (53), Jiang et al. (54), Bonsu et al. (55), Oah et al. (56)
	Individual factors	Individual characteristics	Individual age	Wang et al. (17), Nouri et al. (18)
			Work experience	Chen et al. (19), Wang et al. (17), Nouri et al. (18)
			Personality traits	Baba et al. (20), Sing et al. (21)
		Psychological factors	Safety awareness	Yu et al. (9), Wang et al. (22)
			Safety attitude	Li et al. (11), Tam et al. (23), Li et al. (24), Li et al. (25)
			Work stress	Seo et al. (14), Wu et al. (26)
			Risk perception	Chen et al. (27), Man et al. (28)
		Physiological factors	Fatigue	Kapp et al. (29), Duma (30), Fang et al. (31), Ren et al. (32), Duan (33)
			Insomnia	Kao et al. (34)

### Research Method on the Formation Mechanism of Coal Miners' Unsafe Behavior

The term “mechanism” refers to the relationship and interaction between the elements in a system. The formation mechanism of coal miners' unsafe behavior refers to the relationship and interaction mechanism among the influencing factors of coal miners' unsafe behavior. To study the formation mechanism of coal miners' unsafe behavior, scholars placed workers at the center and explored various influencing factors around them. As the methods adopted by the scholars to explore the formation mechanism of coal miners' unsafe behaviors are different, this study divides and analyzes the formation mechanism of coal miners' unsafe behaviors in detail from the perspective of research methods.

#### SD (Systems Dynamics) Model

SD model is a research method to explore the non-linear causes of behavior in a complex system under feedback and complex variable combination, which can improve the comprehensive understanding of the complex system. It focuses on feedback structure and resulting behavior and has applied in the unsafe behavior of miners (72). Jiang et al. (73) regarded safety management as a system and established an SD (Systems Dynamics) model to identify the causes of workers' unsafe behaviors. They pointed out that safety and production actually support each other, management conditions at the supervision level are effective in improving workers' safety awareness, and preventive actions are more effective than reactive actions in improving safety performance. Yu et al. (9) established an index system of influencing factors of coal miners' unsafe behaviors and reordered the identified influencing factors by using ANP and SD model to determine the key influencing factors. Ma et al. (74) also analyzed the interaction and causality between social factors and individual risk perception by using an SD model and classified multiple feedback loops.

#### Game Theory Model

Game theory is a research method that uses mathematical models to describe the strategic interaction between independent players. It provides optimal decision-making strategies for game players by simulating the situation in the model and has been applied in studying the formation mechanism of coal mine workers' unsafe behaviors (75). Yu et al. (76) studied the symmetry of behavioral benefits of coal miners by using game theory and reported that the stability of safety behavior can be improved by adjusting the symmetry of income between managers and workers through dynamic incentives. Wang et al. (77) asserted that a dynamic game system under flexible cost (incentive and reward) and flexible punishment mechanism can significantly reduce the dynamics of unsafe behaviors in coal mine safety supervision. Similarly, Liu et al. (78) used the evolutionary game model to analyze how the responsibilities of government regulatory departments and inspection of coal mining enterprises affect the efficiency of safety management as well as the role of constraints and incentives in safety management. They also optimized the occupational safety compliance control system of coal mining enterprises by using the game method. Yang et al. (37) established a signal transmission game between miners and managers based on the game theory model to test the influence of managers' emotions on miners' behaviors. Meng et al. (75) reviewed relevant articles on game theory and safety management during 2010–2019 and affirmed that the application of game theory in safety management research is conducive to determine the causes and motivations of unsafe behaviors. Li et al. (79) also discussed the use of evolutionary game theory to describe the interaction between stakeholders in China's coal mine safety production system, including organizations, front-line miners, and front-line managers. They found that when certain conditions are met, the decision-making behaviors of organizations, miners, and managers can evolve into a unique ideal steady state.

#### Structural Equation Model

Structural Equation Model (SEM) is a research method to study the complex relationship between multiple variables by analyzing multivariate data, which is often applied in various fields to explore the relationship between influencing factors (57). SEM has been used to analyze the formation mechanism of unsafe behaviors of coal miners. To improve the intervention effect of behavioral intervention on reducing unsafe behavior of miners, Tong et al. (52) proposed a targeted unsafe behavior intervention method and evaluated the effectiveness of targeted intervention nodes by using an SEM. Liu et al. (10) used an SEM to analyze the hierarchical structure relationship of the HFACS-CM (Human Factor Analysis and Classification System for Coal Miners) model, classified and verified the types of factors that affect miners' unsafe behaviors, and revealed their significant influence on miners' unsafe behaviors. Cheng et al. (80) tested the hypothesis of the influence of leadership behavior on miners' safety behavior by using an SEM. They identified that negative leadership behavior was significantly negatively correlated with miners' safety behavior and its influence was greater than that of positive leadership behavior.

The main research methods mentioned above have their advantages and disadvantages in analyzing the formation mechanism of unsafe behaviors of coal miners, as shown in Table 7. In addition to the above main research methods, scholars also used other research methods to study the formation mechanism of the unsafe behavior of coal miners. For example, Tong et al. (81) proposed a theoretical framework for the analysis of unsafe behavior characteristics based on the analysis of unsafe behavior and the study of miners' pan-scenario data, which could help ascertain the distribution rules of unsafe behavior dimensions and the interaction between different dimensions. Wang et al. (17) used principal component analysis (PCA), binary logistic regression (BLR), and Poisson regression (PR) to analyze the factors influencing miners' safety behaviors and the formation process of unsafe behaviors. Owing to the complexity of these methods, only few studies have been conducted based on these methods.

**Table 7:** The advantages and disadvantages of the main research methods on the formation mechanism of unsafe behavior of miners.

**Research method**	**Advantage**	**Disadvantage**	**Application example**
SEM	It can analyze complex causal relationships among multiple variables. The analysis results have a high degree of visualization, intuitively showing the complex path relationship.	It isn't easy to construct the pre-set conceptual model. The cross-sectional data used can only reflect the situation at that time; Higher requirements on sample size.	Liu et al. (10), Tong et al. (52), Cheng et al. (80)
SD model	Able to simulate and analyze high-order nonlinear complex systems that undergo multiple feedback changes with time. The model can accommodate thousands of variables. Not only rely on data to study system behavior, but also have few requirements on data.	The analysis accuracy of this model is not high. It cannot obtain the optimal solution; Establishing an information feedback mechanism is relatively complicated.	Liu et al. (10), Jiang et al. (73), Ma et al. (74)
Game theory model	This model can analyze the conflicts of interest and cooperation formed by various roles in life. Mathematical models analyze the problems studied, making the analysis process and result more accurate and objective. Able to diagnose complex behavior problems that are difficult to be solved by other methods.	There are too many Nash equilibria to provide unique solutions. The decision-makers in the model are rational and ignore the influence of the surrounding environment and public opinion.	Yang et al. (37), Yu et al. (76), Wa et al. (77), Liu et al. (78), Meng et al. (75), Li et al. (79)

After the induction and analysis of literature on the formation mechanism of the unsafe behavior of coal miners, it is evident that most previous studies used SEM to test the hypothetical relationship between different factors, followed by the game theory model and SD model to carry out a systematic simulation of the dynamic evolution process of unsafe behavior. These studies have discussed the formation mechanism of coal miners' unsafe behavior from different angles, which is helpful in strengthening the understanding of coal mine safety management personnel to the formation mechanism of coal miners' unsafe behavior and can provide guidance for safety pre-control measures.

### Pre-control Method of Coal Mine Workers′ Unsafe Behavior

Based on the influencing factors and forming mechanism of coal miners' unsafe behaviors, corresponding measures should be taken to pre-control unsafe behaviors. Cao et al. (4) suggested that it is imperative to create appropriate management strategies to prevent and control workers' unsafe behaviors in coal mine safety management, and the application of information technology can effectively improve the real-time monitoring and effectiveness of the control of unsafe behaviors. In addition, Jiang et al. (73) reported that management conditions at the supervision level are effective in improving workers' safety awareness, and preventive actions are more effective than reactive actions in improving safety performance. Choudhry et al. (49, 50) also confirmed the importance of safety management by analyzing the influencing factors and formation mechanism of workers' unsafe behaviors and proposed to control workers' unsafe behaviors from two aspects: internal management and information technology. Given this, this study analyzes the previous methods of pre-controlling the unsafe behavior of miners from two aspects of organization management and information technology.

#### Organizational Management

Organizational management is the most direct and effective method to prevent and control the unsafe behaviors of coal miners. It is a combination of internal and external management and managers' leading behaviors (82). Harsini et al. (83) proposed that effective safety leadership, management of safety climate, and culture system are effective measures to intervene in the unsafe behaviors of workers.

Safety management measures can not only improve working conditions, but also positively influence employees' attitudes and behaviors regarding safety and reduce workplace accidents (84). Regarding organizational management, the most effective measure to control the unsafe behaviors of coal miners involves strengthening the internal and external safety management of the organization (85). Li et al. (11) reported that measures, such as establishing a good team safety climate, strengthening safety commitment of managers, providing adequate safety equipment, timely communication with miners, and strengthening safety education and training, can improve the safety attitudes and safety behaviors of coal miners and prevent coal mine accidents. Moreover, Harsini et al. (86) studied the impact of safety education intervention on workers' safety behavior and proposed a mixed recommendation method and virtual training method to conduct safety education and training for workers, proposing that these methods can effectively improve workers' unsafe behavior. Fang et al. (74, 87) affirmed that the implementation of strict safety management and regular safety education and training can affect different aspects of workplace safety climate and effectively regulate workers' unsafe behaviors. Some scholars have also confirmed the usefulness of safety training courses provided by enterprises for workers through research (71).

The good leadership of coal mine enterprise managers can effectively improve the unsafe behavior of miners. Zohar et al. (53) studied the effect of leadership supervision measures on safety behavior and advocated that leaders should maintain safety-oriented interactive feedback with subordinates to improve safety atmosphere and worker safety behavior. Jiang et al. (54, 88) also pointed out that transformational safety leadership strengthens the relationship between safety knowledge and safety participation, whereas passive safety leadership weakens the relationship between safety motivation and safety participation. Managers adopting transformational proactive leadership can make intervention measures on workers' unsafe behaviors more effective. Oah et al. (56) studied the relationship between employees' safety compliance behavior and supervisory leadership practice and found that risk perception related to unsafe behaviors and accidents can be reduced by conducting various safety programs, strengthening managers' leadership ability, and adjusting workers' working speed and workload. Cheng et al. (80) identified that negative leadership behavior is significantly negatively correlated with miners' safety behavior, and its influence is greater than positive leadership behavior. Therefore, leadership incentive measures can be adopted to guide workers in developing effective safety behaviors.

As evident from previous studies, in terms of organizational safety management, managers are inclined to take measures, such as safety education and training, strengthening supervision, creating a good organizational atmosphere, and changing leadership styles, to pre-control the unsafe behaviors of coal miners.

#### Application of Intelligent Technology

With the development of information technology, various technologies have been employed in the mining industry to control workers' behavior. Most studies propose effective pre-control methods through the identification of workers' unsafe behavior and the application of intelligent technology in coal mine safety management.

##### Position Tracking and Behavior Recognition

Wang et al. (89) proposed a prototype system for real-time positioning and tracking of coal miners based on a self-organizing sensor network with higher positioning accuracy and adaptability to understand the position of underground personnel in real time. Guo et al. (90) suggested a real-time control method based on intelligent video surveillance, which can help control the violations of construction personnel through real-time monitoring and analysis, and promote the safety management of construction behavior. Qian et al. (91) proposed an adaptive construction and update method based on a quantum-behaved particle swarm optimization–user-location trajectory feedback (QPSO–ULTF) for a radio fingerprint database, which has a good positioning effect and can ensure the normal operation of the personnel positioning system. Sun et al. (92) suggested that wearable sensors can monitor workers' individual behaviors, measure workers' psychological state by collecting physical and psychological data of on-site workers and identifying workers' personality characteristics related to unsafe behaviors.

##### Big Data Technology

Zhao et al. (93) used the available hidden danger big data to establish a hidden danger quantity prediction model based on a gray neural network, which can better predict the hidden danger quantity of coal mines. Wu et al. (94) built a coal mine safety risk prediction system based on a big data analysis platform. The system dynamically displays coal mine safety production, mining, coal mine accident rules, and predicts and displays coal mine accident risk. This would help in effectively improving the risk prevention, control, and decision-making efficiency of safety supervision departments and coal enterprises. Liu et al. (95) also proposed a recursive neural network relationship extraction method based on a bidirectional minimum gating unit (MGU) model to extract big data in the field of coal mine safety.

##### Virtual Reality Technology

Tang et al. (96) established a virtual miner model with decision-making ability and realized the complex behavior control of virtual miners' multi-agents and interactive simulation of multi-agents in the virtual environment of a coal mine by using object-oriented technology on PC. Grabowski et al. (97) found that the use of VR technology can enable miners to acquire and practice correct behaviors in a controlled and safe environment. They also verified the effectiveness of safety education and training for miners using this technology. Li et al. (98) used a fuzzy evaluation method to quantify the behavior safety of controlled virtual humans in digital simulation, proposed a virtual training method to reduce the occurrence probability of unsafe behavior, developed safety training simulation software, and verified the feasibility of the proposed method through a hypothetical case. Li et al. (99) designed a VR system for coal miners' training based on cloud technology and introduced game artificial intelligence (AI) into the system to increase the emotional communication between the system and users. Rozmus et al. (100) combined 3D laser scanning technology with computer simulation in a CAD/MBS (Computer Aided Design/Multi-Body Simulation) system and used VR technology to visualize the results, proving that this method can improve the safety of miners.

##### AI Technology

Guo et al. (101) proposed a bone-based real-time recognition method combining various technologies, which can simplify dynamic movement into static posture to identify unsafe behaviors. Nguyen et al. (102) used Vietnam as an example and established four AI models, including artificial neural network (ANN), k-nearest neighbor (KNN), support vector machine, and classification regression tree. They ascertained that the advanced computational model was much better than the empirical method for estimating the ground vibration caused by blasting. Nie et al. (103) established a computational mine safety supervision model based on multi-agent modeling and simulation technology and resource conservation technology. They also made suggestions to improve the mine safety supervision system and provided an improved safety management decision basis for reducing the occurrence of mine accidents. Bui et al. (104) applied seven AI methods to predict specific blasting-induced AOp, including random forest, support vector regression, Gaussian process, Bayesian additive regression tree, enhanced regression tree, KNN, and ANN, and pointed out that AI technology is excellent for predicting blasting-induced AOp in open-pit mines.

##### Internet of Things Technology

Zhou et al. (105) proposed a safety barrier early warning system based on IoT, which provides safety barrier strategies and scenarios to avoid unsafe behaviors and unsafe states of construction equipment and worker environment. Singh et al. (106) identified that IoT could play an important role in mining operations by improving worker safety and productivity. Jo et al. (107) integrated a high-precision FBG (Fiber Bragg Grating) monitoring system and an output-only data-driven method on the IoT platform to develop a comprehensive mine structure safety system. Practical applications show that the integration of FBG technology and IoT can be effectively utilized for early safety evaluation and real-time information sharing in underground coal mines. Xie et al. (108) proposed an adaptive heuristic mathematical model based on IoT and RFID (Radio Frequency Identification) real-time monitoring system, which allows real-time tracking, detection of suspicious events, and verification of the location of miners in harsh underground mining environments. Wu et al. (109) established a dynamic information platform for underground coal mines based on IoT. The establishment of this platform can greatly improve users' ability of hazard identification and follow-up decision-making and ensure the safety of underground mining.

Various intelligent technologies have been widely applied in the field of coal mine safety management. In particular, VR technology is used to conduct safety education and training for coal miners and IoT technology is used to identify the unsafe behaviors of coal miners, so that the unsafe behaviors of coal miners are well controlled. The application of various intelligent technologies in coal mine safety management to build a comprehensive management platform for coal mine safety has become a trend. With the progress of science and technology, the promotion of intelligent technology and equipment, and the continuous optimization of coal mining technology, the behavior of coal miners can be better controlled and the incidence of unsafe behavior would decrease gradually.

## Results and Discussion

Based on the systematic literature review, this study summarizes and discusses the previous literature, and proposes the future research direction in the topic of “unsafe behavior of coal miners.”

As for the research on the influencing factors of coal miners' unsafe behaviors, most scholars discuss the influence factors on individual miners' unsafe behavior from personal and organizational management. However, results based on the individual level of workers' unsafe behavior cannot reflect the overall situation of unsafe behavior of coal miners. Therefore, it is necessary to explore the factors influencing the unsafe behavior of coal miners at the group level and the improvement of group behavior. In future research, scholars can use various methods to explore the unsafe behavior of the miners' group. For example, grounded theory can explore the factors affecting miners' unsafe behaviors, and group dynamics theory can analyze the interaction between miners' groups and workers' unsafe behaviors (110, 111).

As for the formation mechanism of coal miners' unsafe behavior, the current research mainly focuses on building models from one or more aspects of individual factors, organizational management factors, and environmental factors to analyze the formation mechanism of individual unsafe behavior. However, the entire formation process of coal miners' unsafe behavior is complicated, and it is difficult to explain the complete evolution process using only SEM, SD model, or game theory model. Therefore, in future research, a mixture of various models and intelligent technologies can be considered to establish an unsafe behavior analysis system to systematically analyze the formation mechanism of the unsafe behavior of coal miners. For example, scholars can use multi-agent modeling, complex adaptive system modeling, and BP neural network to build an analysis system for coal miners' unsafe behavior. The system can analyze the formation mechanism of the unsafe behavior of individuals and groups of miners and predict the unsafe behavior of workers (85, 112).

As for the pre-control method for coal miners' unsafe behaviors, the most common method adopted by managers is safety education and training for workers, which is also considered to be the most effective intervention measure to improve workers' unsafe behaviors (11, 84). However, owing to the mandatory safety education and training methods and boring training content, this method does not actually improve the unsafe behavior of workers. Therefore, from the perspective of workers' needs and the purpose of safety education of enterprises, training methods with personalized recommendation functions and interactive communication with workers can be used to improve workers' subjective initiative and the effectiveness of safety education and training, while making the training content more interesting (12). In addition, coal mining enterprises often introduce various technologies and equipment to supervise and control workers' unsafe behaviors (92, 113). However, the unsafe behavior of miners is the result of the interaction between their behavior environment and self-regulation (114, 115). It is challenging to pre-control the unsafe behavior of miners only by monitoring. Therefore, from the psychological and physiological perspective of miners, managers of coal mine enterprises can take various measures to improve the behavior environment of miners and fundamentally control the unsafe behavior of workers.

## Conclusions

The unsafe behavior of coal miners has attracted the attention of scholars in the field of coal mine safety management. This paper uses a systematic literature review to classify and analyze the previous research on coal mine workers' unsafe behavior. The results show that the previous research topics mainly include the influencing factors, formation mechanism, and pre-control measures of unsafe behavior of miners. In terms of influencing factors, personal factors, organizational management factors, and environmental factors are the main factors that affect the unsafe behavior of miners. In terms of research methods, SEM, SD model, and game theory model are the most frequent methods scholars use to analyze the formation mechanism of mine insecurity. In terms of pre-control methods, most scholars put forward measures to intervene in the unsafe behavior of miners from the perspective of organizational management and intelligent technology. In the past, research on coal mine workers' unsafe behavior was rich and varied, but there are still some deficiencies. Because of this, this study puts forward the future research direction from the following three aspects.

First, enrich the research object of coal mine workers' unsafe behavior. The unsafe behavior of coal mine workers includes individual behavior and group behavior. The previous research mainly focused on the unsafe behavior of individual workers and ignored the influence of the group of workers on the unsafe behavior. Therefore, future research can strengthen the study of unsafe behaviors of workers' groups. In addition, it is necessary to enhance the research on the unsafe behavior of workers in different types of work because of the various manifestations of unsafe behavior of workers.

Second, introduce innovative research methods that can systematically analyze the formation mechanism of unsafe behaviors of miners. The formation of coal mine workers' unsafe behavior is a complex and dynamic process that needs to be examined by systematic thinking and methods and is challenging to realize by traditional research methods. Therefore, it's necessary to consider a more comprehensive innovative research method to systematically analyze the formation mechanism of coal mine workers' unsafe behavior from multiple perspectives.

Third, introduce various psychological theories to study the unsafe behavior of miners. The formation of miners' unsafe behavior results from the interaction between their behavior environment and self-regulation, which involves many psychological factors and needs in-depth discussion by scholars. Previous researches are more about the behavior environment of miners but less about the unsafe behavior of miners from the perspective of psychology. Unsafe behaviors of miners are often the external manifestation of complex psychological activities, so analyzing their unsafe behaviors from a psychological standpoint can fundamentally understand the causes of unsafe behaviors and take adequate measures to prevent and control unsafe behaviors.

Last, strengthen the research of coal mine workers' physiological and psychological monitoring technology. At present, the monitoring technology and equipment used in coal mine enterprises control the unsafe behavior of workers by visually monitoring their external behavior. However, the technology and equipment to monitor miners' psychological and physical indicators to reflect their unsafe behavior are still lacking. Therefore, future research needs to increase investment in this area.

This study provides a valuable basis and inspiration for coal mine safety managers to improve coal mine safety management in practice and has practical guiding significance in this field. Given the previous research on the unsafe behavior of miners, this paper puts forward some suggestions on the safety management of coal mine enterprises from two aspects of control measures and supervision.

According to the causes of the unsafe behavior of miners, Coal mine managers should control the unsafe behavior from the following aspects. (1) Establish a safe atmosphere to guide the workers to establish safety awareness. (2) Strengthen safety education and training to enrich workers' safety knowledge and skills. (3) Strengthen supervision to restrain worker behavior. (4) Improve the working environment to make workers comfortable. (5) Strengthen internal and external communication within the organization to solve all kinds of problems workers encounter. (6) Positive incentive measures increase workers' enthusiasm to avoid unsafe behaviors.

Coal mine managers can take the following measures to supervise and manage the unsafe behaviors of miners. (1) Establish and improve the system of discovering, reporting, and representing workers' unsafe behaviors to realize the standardized supervision of unsafe behaviors. (2) Improve the teaching and assessment system of unsafe behavior to recognize the professional intervention of unsafe behavior. (3) Establish a system of returning to post and visiting workers for unsafe behavior to avoid the recurrence of unsafe behavior. (4) Conduct statistics and analysis of workers' unsafe behaviors to formulate effective control measures. (5) Establish and improve the responsibility and accountability system for workers' unsafe behavior to punish workers who have shown unsafe behavior.

## Data Availability Statement

The data set provided in this study can be found in two databases, Web of Science and PubMed, and the method of obtaining it has been described in detail in the Literature Source section of the article.

## Author Contributions

XW designed and conceptualized the study and wrote the manuscript. LY supervised the project and obtained funding. XW, JZ, and ZQ download the related papers and also obtained funding. All authors participated in screening the articles and provide critical feedback, significantly contributed to the study, and approved the final manuscript.

## Funding

This study was supported by the National Natural Science Foundation of China under the grant (No. 71971003), the Major of National Social Science Foundation of China (No. 20ZDA084), and the Graduate Innovation Fund Project (No. 2020CX1009).

## Conflict of Interest

The authors declare that the research was conducted in the absence of any commercial or financial relationships that could be construed as a potential conflict of interest.

## Publisher's Note

All claims expressed in this article are solely those of the authors and do not necessarily represent those of their affiliated organizations, or those of the publisher, the editors and the reviewers. Any product that may be evaluated in this article, or claim that may be made by its manufacturer, is not guaranteed or endorsed by the publisher.
